# Comparison of the psychometric performance of experimental EuroQol Toddler and Infant Populations Instrument (EQ-TIPS) and Pediatric Quality of Life Inventory™ (PedsQL) in young children

**DOI:** 10.1007/s10198-025-01804-4

**Published:** 2025-07-30

**Authors:** Tianxin Pan, Li Huang, Vicky He, Janine Verstraete, Nancy Devlin, Mike Herdman, Kim Dalziel, in collaboration with the Quality Of Life in Kids: Key evidence to strengthen decisions in Australia (QUOKKA) project team

**Affiliations:** 1 Child Health Economics Unit, Centre for Health Policy, Melbourne School of Population and Global Health, The University of Melbourne, VIC, Australia; 2 Department of Paediatrics and Child Health, Division of Medicine, University of Cape Town, Cape Town, South Africa; 3 Saw Swee Hock School of Public Health, National University of Singapore, Singapore, Singapore; 4 Health Services and Economics, Murdoch Children’s Research Institute, Melbourne, VIC Australia

**Keywords:** Psychometric performance, Children, EQ-TIPS, PedsQL, Infants, Health-related quality of life

## Abstract

**Objective:**

This study aimed to test and compare the validity, reliability and responsiveness of the Experimental EuroQol Toddler and Infant Populations version 2.0 (EQ-TIPS) and the Paediatric Quality of Life Inventory™ version 4.0 (PedsQL) in young Australian children.

**Methods:**

Data for children aged 2–3 years were available from the Australian Paediatric Multi-Instrument Comparison (P-MIC) study. EQ-TIPS and PedsQL were administered in randomised order to caregivers of children aged 2–3 years. We assessed acceptability, feasibility, response distribution, ceiling effects, known-group validity, convergent validity, test-retest reliability and responsiveness in the total sample and by child age. EQ-TIPS level sum scores and PedsQL total scores were used as outcomes.

**Results:**

A total of 510 caregivers of children completed the initial survey and 286 completed a follow-up survey at four weeks. The EQ-TIPS had ceiling effects in children with special health care needs (25.8%). EQ-TIPS showed stronger effect sizes in differentiating children with health conditions whereas PedsQL showed statistical significance and a minimal clinically important difference in detecting proxy-reported improvements in health. We found moderate to strong correlations in all anticipated EQ-TIPS and PedsQL item pairs. The two instruments showed comparable performance in children age 2 and age 3.

**Conclusion:**

Both EQ-TIPS and PedsQL are valid instruments, with small differences in psychometric performance depending on user needs and context. EQ-TIPS was easier to complete and had better known-group validity and test-retest reliability, whereas PedsQL was more sensitive in detecting health improvements. Further research on the responsiveness of EQ-TIPS is needed.

**Supplementary Information:**

The online version contains supplementary material available at 10.1007/s10198-025-01804-4.

## Introduction

Patient-reported outcome measures (PROMs) are widely used in clinical trials, population health monitoring, and economic evaluations to inform healthcare resource allocation and decision-making. Children are important users of healthcare services and resources, with those under five years being particularly vulnerable to illness. In 2019, children under five years accounted for nearly 20% of the global burden of disease [1]. Among the known rare diseases, 75% affect children [2]. These conditions pose substantial disease and economic burdens to families and society. There are increasing effort in implementing and evaluating new technologies such as genomics and precision medicine for affected children [3, 4], and growing interest in measuring health-related quality of life (HRQOL) among children under five years.

However, there are few options for measuring HRQOL in very young children. Until very recently there was a complete absence of preference-weighted measures developed or validated in children under five years, which limited health economic evidence of interventions targeting this vulnerable age group. While the Paediatric Quality of Life Inventory ^TM^ version 4.0 (PedsQL) is a widely used PROM in children under five years, it lacks a means of preference weighting its data. Recently, two research teams have shortened PedsQL to develop a health state classification systems (HSCS) for eliciting preference for use in children 2 years and above [5, 6]. However, research is ongoing and no scoring algorithms are available yet. In addition, the two HSCS for PedsQL comprise quite different items and further research is therefore needed to understand these differences and their implications for QALY estimates generated from them. There are clear advantages to concise, generic preference-accompanied measures - demand for which has led to the development new paediatric PROMs for younger children, including the Infant Health-Related Quality of Life Instrument (IQI) for infants aged 0–12 months [7], the Health Utilities Preschool (HuPS) [8], and the EuroQoL Toddler and Infant Populations (EQ-TIPS) measure (previously known as TANDI) for children aged 1–36 months [9]. Other instruments, whilst not specifically created for young children, have adapted versions such as the CHU9D [10] and the EQ-5D-Y with guidance notes for 2–4 years [11].

The EQ-TIPS was developed in English in South Africa specifically for infants and toddlers [9]. It was developed with a view of broadly aligning with the EuroQol’s HRQoL instruments for children and adolescents (i.e. EQ-5D-Y-3L and EQ-5D-Y-5L) [12, 13], in terms of instructions, layout, recall period, level descriptors, and inclusion of the EQ VAS. It is currently considered an experimental instrument by the EuroQol Group, which implies that it is still under development [14]. The detailed development process of EQ-TIPS has been described elsewhere [9]. To date, evidence on the content validity and psychometric performance of the EQ-TIPS was mostly from South Africa [15–19]; with one recently published study examining the performance of EQ-TIPS in children with COVID-19 in China [20]. Further validation of the EQ-TIPS in other countries is needed to better understand its psychometric properties and inform its use.

The PedsQL toddler version for 2–4 years has been widely used in different clinical conditions, with some applications in general toddler population worldwide [21–23]. However, evidence on the validity of PedsQL in 2–4 years is relatively limited in Australia where only one recent study validated its use in 2-4-year-old Australian children [10].

This study aimed to compare the validity, reliability and responsiveness of the experimental EQ-TIPS version 2.0 and the PedsQL among young Australian children both from the general population and with health conditions.

## Methods

### Data and sample

This study used a subset of data collected via the Australian paediatric multi-instrument comparison (P-MIC) study (data cut 2, dated 10 August 2022) [24]. The P-MIC study aimed to generate a comprehensive database that includes various paediatric PROMs to understand their performance. Participants included children aged 2–18 years and their primary caregivers. Participants were recruited onsite through the Royal Children’s Hospital Melbourne and online via a market research company, Pureprofile, in Australia. All participants consented to participate in the P-MIC study, completed an initial survey and were asked to complete a follow-up survey at 4 weeks. Ethical approval was obtained via the Royal Children’s Hospital Human Research Ethics Committee (HREC/71872/RCHM-2021). Detailed information on the P-MIC study has been reported elsewhere [24].

In the P-MIC study, for children aged 2–3 years (up to 47 months and 29 days), their primary caregivers (i.e., acting in a caregiving role for the child at the time of completing the survey, such as parents, legal guardian, grandparents) were presented with a series of demographic questions followed by several PROMs including experimental EQ-TIPS version 2.0 and PedsQL core generic version 4.0, which were presented in randomised order using proxy perspective (i.e., assessment of the child’s health status is provided from the caregivers’ perspective).

### Measures

#### Paediatric PROMs

##### EQ-TIPS

The EQ-TIPS was developed for children aged 1–36 months for proxy report. EQ-TIPS experimental version 2.0 includes a descriptive system consisting of six items: Movement, Play, Pain, Communication, Social interaction and Eating, and a Visual Analogue Scale (EQ VAS) on a 0 (worst) to 100 (best) scale. Each EQ-TIPS-3L item has three levels: level 1 (no problems), level 2 (some problems) and level 3 (a lot of problems) [9, 16]. The EQ-TIPS uses the recall period of ‘today’.

The health state described by the EQ-TIPS descriptive system can be summarised as a six-digit string, representing the level of each dimension in the order presented in the questionnaire. The best health state is described as 111111 and the worst 333333. Due to a lack of preference weights, we reported level sum score (LSS) as the summary scores for the descriptive system, which ranges from 6 to 18. A lower LSS suggests better HRQOL. Caregivers were required to answer all EQ-TIPS questions therefore there is no missing EQ-TIPS data.

##### PedsQL core generic version 4.0

The PedsQL core generic version 4.0 for toddlers aged 2–4 years was used in this study [25]. The PedsQL uses the recall period of ‘during the past ONE month’. It includes 21 items across four domains: physical-functioning (8 items), emotional-functioning (5 items), social-functioning (5 items) and school-functioning (3 items). Each item has a raw score ranging from 0 (never a problem) to 4 (always a problem). Following the PedsQL scoring manual, item response is converted to a 0 to 100 scale as follows: 0 = 100, 1 = 75, 2 = 50, 3 = 25, 4 = 0 [26]. Domain level scores were calculated by the summation of item scores (on a 0-100 scale) over the number of completed items in each domain. The total PedsQL score was calculated as the sum of all the items over the number of completed items. A higher PedsQL score suggests better HRQOL.

For children aged 2–4 years, the P-MIC study did not permit missing responses on paediatric PROMs except for PedsQL’s school domain as per PedsQL recommended-administration given that its items may not have been relevant to their children. Following the PedsQL scoring instructions [26], if responses on two or more items in school domain were missing, the domain score were not computed. The total PedsQL score was calculated as the sum of all the items over the number of items answered on all the domains.

#### Caregiver-reported health condition

The P-MIC survey included caregiver-reported questions on children’s general health status, special healthcare needs and the presence of a wide range of health conditions.

##### General health status

The general health question asked the caregivers to describe their child’s general health today as ‘excellent’, ‘very good’, ‘good’, ‘fair’ or ‘poor’. Due to a skew towards better health, the ‘fair’ and ‘poor’ categories were combined for analysis. Children’s general health status was used to analyse the distribution of responses and in the known-group analysis.

##### Special healthcare needs (SHCNs)

Children were identified as having special healthcare needs (SHCNs) based on a validated screening questionnaire if requiring more than average use of health services over a 6-month period [27]. They were categorised as with or without SHCNs, and this categorization was used in the known-group analysis.

##### Health condition groups and healthy reference group

In the survey, caregivers were asked to report any doctor-diagnosed health conditions of their child from a list of 43 common conditions available from the Longitudinal Study of Australian Children and the literature [28, 29], as well as a few rare conditions we have targeted to recruit. The health conditions that most commonly occurred (with > 5% prevalence per condition group for age 2–3 years in this study) were used for known group analysis.

To maximise sample size for analysis, we grouped asthma, allergies, eczema and hay fever under ‘atopy’, and grouped developmental delay, Attention Deficit/Hyperactivity Disorder (ADHD), Autism, behavioural, cognitive and emotional problems as ‘neurological conditions’. A healthy reference group was defined as those without health conditions and reporting an EQ VAS score of at least 70 at baseline [30].

##### Changes in health status

In the follow-up survey 4 weeks after, caregivers were asked to report their child’s change in health. Specifically, the survey asked, “How would you rate the Study Child’s health in general now?” with response options as “(1) much better, (2) somewhat better, (3) about the same, (4) somewhat worse, or (5) much worse”. In addition, caregivers who reported their children with a health condition in the initial survey were asked “Thinking about the Study Child’s main health condition, how would you say this is going now compared to when you completed the first survey for this study?”. The response options were the same as those used for change in general health. Responses to these questions were used to identify if children had changes in health status and to identify the sample for test-retest reliability (if reported ‘about the same’) and responsiveness tests for improvements in health (if reported ‘much better’).

#### Socio-demographic measure

Socio-demographic characteristics, including children’s age, gender, ethnic group, language spoken at home, caregiver’s educational background and household income were reported by parents.

### Data analysis

This study predominantly follows the psychometric analysis plan listed in the P-MIC study technical methods paper [30]. We examined acceptability and feasibility, response distributions including ceiling and floor effects, known-group validity, convergent validity at baseline, and test-retest validity and responsiveness using baseline and follow-up data. Analyses were performed using R (version 4.3.1).

#### Acceptability and feasibility

Acceptability and feasibility were assessed descriptively by presenting the time taken to complete each instrument (in seconds) and the reported difficulty in completing each instrument. Reported difficulty was asked after each instrument, rated on a 1 (very difficult) to 5 (very easy) scale.

#### Response distributions & ceiling and flooring effect

At the item level, we reported response frequencies of each level of EQ-TIPS items and compared the differences across the proxy-reported general health status (excellent, very good, good and fair/poor) using Fisher’s exact statistics. We reported the response distribution of PedsQL items and plotted the four domain scores of PedsQL. At the instrument level, we plotted the distribution of EQ-TIPS LSS, EQ VAS score and PedsQL total score for the total sample and by general health status.

The ceiling and floor effects were assessed by reporting the proportion of no problems (111111) or a lot of problems (333333) across all dimensions for EQ-TIPS, and by reporting the proportion of a score of 100 or a score of 0 on PedsQL total scores. Given that ‘no problems’ is likely to be a genuine response for most children in the general population sample, ceiling effects were assessed in subgroups of children with proxy-reported poor or fair health and SHCNs. Following literature, a 15% ceiling or floor effect threshold was used [31].

#### Known group validity

Known-group validity assesses the sensitivity of instruments to detect the differences between groups where responses might be expected to differ. Group differences were assessed by comparing the mean instrument total scores (EQ-TIPS LSS and PedsQL total scores respectively) among groups. The statistical significance of the difference was tested using nonparametric Mann–Whitney U test or Kruskal-Wallis test as the summary scores were not normally distributed. The magnitude of the difference was assessed using Cohen’s D effect size. Effect sizes of 0.2–0.49, 0.5–0.79 and ≥ 0.8 were considered small, moderate, and large respectively [32, 33]. A mean difference with a *p* value of < 0.05 and a large effect size (≥ 0.8) was considered good performance. We compared the known-group validity across the EQ-TIPS and PedsQL for a number of subgroups including general health status (fair or poor, very good, good health each compared to excellent health), children with SHCNs (yes/no) and three most prevalently reported condition (sleep problems, atopy, neurological conditions) compared to a healthy reference group (as described in 2.2.2). It was expected that children with proxy-reported fair or poor health, those with SHCNs and with health conditions would have poorer HRQOL compared to their healthier counterparts. In addition, we explored the presence and magnitude of the differences between ages 2 and age 3 years. We hypothesised that there would be no age-related differences in HRQOL scores.

#### Convergent validity

Convergent validity is evaluated by examining the extent to which a specific item of the instrument demonstrates significant correlations with items that are theoretically expected to be related. Correlations were calculated using Spearman’s correlation. Correlations of 0.1–0.29 were considered weak, 0.3–0.49 moderate, and ≥ 0.5 strong [33]. We hypothesised a moderate-to-strong correlation between EQ-TIPS LSS and PedsQL total score [15, 18]. At the item level, we hypothesised that moderate-to-strong correlations would be identified between items from the following EQ-TIPS and PedsQL item pairs: Movement vs. ‘Walking’ and ‘Running’; Play vs. ‘Participating in active play’, ‘Exercise and playing with other children’ and ‘Keeping up when playing with other children’; Pain vs. ‘Having hurts or aches‘; Social interaction vs. ‘Playing with other children’.

#### Test-retest reliability

Test-retest reliability assesses the stability of the scores generated by an instrument by the same person between two time points, assuming no changes in health status between the two time points. Children whose caregivers completed the 4-week follow-up and reported “about the same” on the general health change were included in this analysis. We assessed test-retest reliability of EQ-TIPS items using Cohen’s weighted kappa coefficients for the subgroup of participants who reported no change in the child’s general health or health condition. Coefficient values of 0.2 were considered poor, 0.21–0.40 fair, 0.41–0.6 moderate, 0.61–0.80 substantial and > 0.81 almost perfect agreement, respectively [34]. For EQ-TIPS LSS, PedsQL domain and total scores, we used intraclass correlation coefficient (ICC) estimates and reported corresponding 95% confidence intervals. ICC estimates were calculated based on an absolute-agreement, two-way mixed-effects model. ICC values of < 0.4 were considered poor, 0.4–0.59 fair, 0.60–0.74 good, and ≥ 0.75 excellent reliability, respectively [35].

#### Responsiveness

Responsiveness assesses an instrument’s ability to detect changes in health over time. We assessed responsiveness at the instrument level, by comparing the differences in mean instrument total scores between the initial and follow-up surveys. The magnitude and statistical significance of the difference were assessed using Standardised Response Mean (SRM) effect size statistic and paired t-test, respectively. The SRM effect size was calculated as the ratio of the mean change of instrument total scores to the standard deviation (SD) of the changes in scores. The values of 0.2–0.49, 0.5–0.79, and ≥ 0.8 were considered small, moderate, and large effect sizes [32, 33]. The PedsQL is the only measure with an established minimal clinically important difference (MCID) which is 4.5 points on the summary score for proxy report [36]). Children whose caregivers reported improvements in general health or main health condition in the follow up survey were included. Due to limited sample size, we were not able to assess responsiveness to worsened health.

#### Subgroup analysis

To provide a comprehensive psychometric comparison of EQ-TIPS and PedsQL in young children, we conducted subgroup analysis by age (aged 2 years and 3 years respectively), and reported response distributions, known-group validity, convergent validity and test-retest reliability for ages 2 and age 3 years.

#### Summary of methods for psychometric assessment

We summarised the psychometric performance of EQ-TIPS and PedsQL and considered good performance based on the following thresholds [37]:

- Response distribution (no ceiling effect): < 15% of participants with SHCNs report full health (111111 on EQ-TIPS and 100 on PedsQL total scores).
- Known-group validity: a mean difference with a p value of < 0.05 and a large effect size (≥ 0.8).
- Convergent validity: moderate to strong correlations (Spearman’s correlation coefficients ≥ 0.3) between items or dimensions hypothesised to be correlated.
- Test-retest reliability: good or excellent agreement (ICC ≥ 0.6).
- Responsiveness: a mean difference with a p value of < 0.05.

## Results

### Sample characteristics

The P-MIC study included responses for 510 children aged 2–3 years in the initial survey, among which 223 (43.7%) were recruited via Royal Children’s Hospital, 248 (48.6%) were general population recruited via online panel and 39 (7.7%) were recruited for specific conditions (all were with sleeping problems in this age range) via online panel. In our sample, there were slightly more males (52.2%) and children aged 2 years (51.6%). The response rate for the follow-up survey at 4 weeks was 286 (56.1%).

In our sample, 31.2% of children were reported to have a SHCN, the most prevalent health conditions were eczema (18.8%), sleep problems (14.7%), asthma (12.0%), and developmental delays (11.2%). Nearly 80% of caregivers reported their children’s general health as excellent or very good, with only 6.1% reported as fair or poor. A total of 173 children were reported as having no change in health between baseline and follow-up, and 43 and 10 reported improved or worsened health, respectively. The characteristics of our sample were largely comparable with the estimates from the national representative study (Longitudinal Study of Australian Children), except that caregivers in our sample had higher education and income (Table 1). Sample characteristics for each general health status groups are reported in ESM Table A1.

**Table 1:** Sample characteristics at baseline

Characteristics	Total sample (*N* = 510)	LSAC^+^
**Sample source (Sample 1**,** 2**,** 3)**		
Sample 1 (children with or without health conditions recruited via a large tertiary paediatric hospital)	223 (43.7%)	
Sample 2 (general population recruited via an online panel)	248 (48.6%)	
Sample 3 (specific conditions recruited via an online panel– for EQ-TIPS sample only includes sleep problems)	39 (7.7%)	
**Child gender**		
Male	266 (52.2%)	51.66
Female	241 (47.3%)	48.34
Other	3 (0.6%)	
**Child age (years)**		
2	263 (51.6%)	
3	247 (48.4%)	
**Aboriginal or Torress Strait Island origin**		
Yes	26 (5.1%)	2.65
No	483 (94.7%)	97.35
Prefer not to say	1 (0.2%)	
**Child speaks English at home**		
Yes	422 (82.7%)	
**Relationship with the study child**		
Parent/legal guardian	494 (96.9%)	
Grandparent	9 (0.17%)	
Carer unrelated to the child	5 (0.10%)	
Siblings or other relatives	2 (0.04%)	
**Caregiver education-bachelor’s degree or above **		
Yes	262 (51.4%)	34.95%
**Single parent household**		
Yes	72 (14.3%)	
**Household weekly income before tax (AUD)**		
Less than $500 per week ($25,999 or less per year)	21 (4.2%)	5.46%
$500-$999 per week ($26,000-$51,999 per year)	87 (17.5%)	16.81%
$1,000-$1,999 per week ($52,000-$103,9799 per year)	188 (37.8%)	48.17%
$2,000 or more per week ($104,000 or more per year)	201 (40.4%)	29.57%
**Remoteness**		
Major cities	388 (76.1%)	
Inner regional	91 (17.8%)	
Outer regional	26 (5.1%)	
Remote/very remote	5 (1.0%)	
**Completed 4-week follow up survey**		
Yes	286 (56.1%)	
**In general**,** how would you say the study child’s current health is?**		
Excellent	198 (38.8%)	52.72%
Very good	196 (38.4%)	34.32%
Good	85 (16.7%)	10.99%
Fair or poor	31 (6.1%)	1.86%
**Child having special health care needs**		
Yes	159 (31.2%)	12.74%
Health conditions with prevalence > 5%*		
Constipation	39 (7.6%)	
Developmental delay	57 (11.2%)	
Eczema	96 (18.8%)	
Food or digestive allergies	51 (10.0%)	
Hay fever	27 (5.3%)	
Asthma	61 (12.0%)	
Behavioural, cognitive & emotional problems	30 (5.9%)	
Sleep problems	75 (14.7%)	
**Healthy reference group (i.e.**,** without any ongoing conditions and having a EQ VAS score of at least 70)**		
Yes	144 (28.2%)	
**Change in general health**		
much better	43 (8.4%)	
somewhat better	60 (11.8%)	
about the same	173 (33.9%)	
somewhat or much worse	10 (2.0%)	
**Change in main health conditions**		
much better	33 (15.9%)	
somewhat better	39 (18.8%)	
about the same	128 (61.5%)	
somewhat or much worse	8 (3.9%)	

All information were collected by parents/caregiver proxy report

*The survey asked about ongoing conditions from a list of 43 [28, 29]. We report all those with prevalence of > 5% for children aged 2/3 years included in this analysis

+ We examined our sample representativeness by comparing statistics estimated from the national representative Longitudinal Study of Australian Children (LSAC). Estimates obtained from Xiong et al. 2024 [10].

### Acceptability and feasibility

The mean (and median, interquartile range IQR) time of completing EQ-TIPS and PedsQL was 48.9 s (27.1, IQR 19.9–39.9) and 152.7 s (86.2, IQR 64.3–120). As expected, the median time of completion and variation increased with the severity grouping according to the caregiver-reported general health status of the child (ESM 1 Table A2). In the total sample, 87% and 80% found it somewhat or very easy to complete EQ-TIPS and PedsQL respectively. Similarly, the poorer the caregiver-reported general health status of the child, the more difficult to complete the questionnaire for both instruments. Across all general health status groups, higher proportions of respondents reported EQ-TIPS easy to complete (81-94%), compared to PedsQL (68 − 90%) (ESM 1 Table A3).

### Response distribution

Table 2 reports the response distribution for EQ-TIPS. The proportions of respondents reporting at least some problems (level 2 or level 3) for Movement, Play, Pain, Social interaction, Communication and Eating were 12.0%, 13.1%, 18.8%, 26.3%, 28.0% and 39.2% respectively. In the poor or fair health group, there were higher proportions of reporting problems in eating (68%), social interaction (55%) and pain (53%). There was significantly higher report of problems across all EQ-TIPS items in children reported as having worse general health (*p* < 0.05).

**Table 2:** Response distribution of EQ-TIPS

Dimension	Total sample (*N* = 510)	General health groups				
		Excellent (*N* = 198)	Very good (*N* = 196)	Good (*N* = 85)	Fair or poor (*N* = 31)	*p*-value (Fisher’s)
**Movement**						< 0.001
Level 1	449 (88.0%)	193 (97.5%)	175 (89.3%)	60 (70.6%)	21 (67.7%)	
Level 2	47 (9.2%)	4 (2.0%)	18 (9.2%)	20 (23.5%)	5 (16.1%)	
Level 3	14 (2.7%)	1 (0.5%)	3 (1.5%)	5 (5.9%)	5 (16.1%)	
**Play**						< 0.001
Level 1	443 (86.9%)	187 (94.4%)	176 (89.8%)	59 (69.4%)	21 (67.7%)	
Level 2	55 (10.8%)	10 (5.1%)	18 (9.2%)	22 (25.9%)	5 (16.1%)	
Level 3	12 (2.4%)	1 (0.5%)	2 (1.0%)	4 (4.7%)	5 (16.1%)	
**Pain**						< 0.001
Level 1	414 (81.2%)	187 (94.4%)	162 (82.7%)	50 (58.8%)	15 (48.4%)	
Level 2	93 (18.2%)	11 (5.6%)	33 (16.8%)	34 (40.0%)	15 (48.4%)	
Level 3	3 (0.6%)	0 (0%)	1 (0.5%)	1 (1.2%)	1 (3.2%)	
**Social** **interaction**						< 0.001
Level 1	371 (72.7%)	173 (87.4%)	144 (73.5%)	40 (47.1%)	14 (45.2%)	
Level 2	109 (21.4%)	21 (10.6%)	44 (22.4%)	34 (40.0%)	10 (32.3%)	
Level 3	30 (5.9%)	4 (2.0%)	8 (4.1%)	11 (12.9%)	7 (22.6%)	
**Communication**						< 0.001
Level 1	367 (72.0%)	172 (86.9%)	137 (69.9%)	42 (49.4%)	16 (51.6%)	
Level 2	103 (20.2%)	22 (11.1%)	45 (23.0%)	28 (32.9%)	8 (25.8%)	
Level 3	40 (7.8%)	4 (2.0%)	14 (7.1%)	15 (17.6%)	7 (22.6%)	
**Eating**						< 0.001
Level 1	315 (61.8%)	158 (79.8%)	114 (58.2%)	33 (38.8%)	10 (32.3%)	
Level 2	147 (28.8%)	38 (19.2%)	67 (34.2%)	35 (41.2%)	7 (22.6%)	
Level 3	48 (9.4%)	2 (1.0%)	15 (7.7%)	17 (20.0%)	14 (45.2%)	
Proportion of reporting full health (111111)	219 (42.9%)	130 (65.7%)	74 (37.8%)	11 (12.9%)	4 (12.9%)	< 0.001
LSS (mean, SD)	7.6 (1.5%)	6.7 (3.4%)	7.6 (3.9%)	9.3 (10.9%)	10.1 (32.6%)	< 0.001
EQ VAS (mean, SD)	82.7 (16.2%)	91.8 (46.4%)	83.4 (42.6%)	72.4 (85.2%)	49.3 (159%)	< 0.001

Level 1: no problems, Level 2: some problems; Level 3: a lot of problems

General health groups were identified based on parents/caregivers response to the general health question to describe their child’s general health today as ‘excellent’, ‘very good’, ‘good’, ‘fair’ or ‘poor’. Based on this question, children were categorized into four groups: (1) excellent, (2) very good, (3) good, (4) fair and poor

**Fig. 1:**
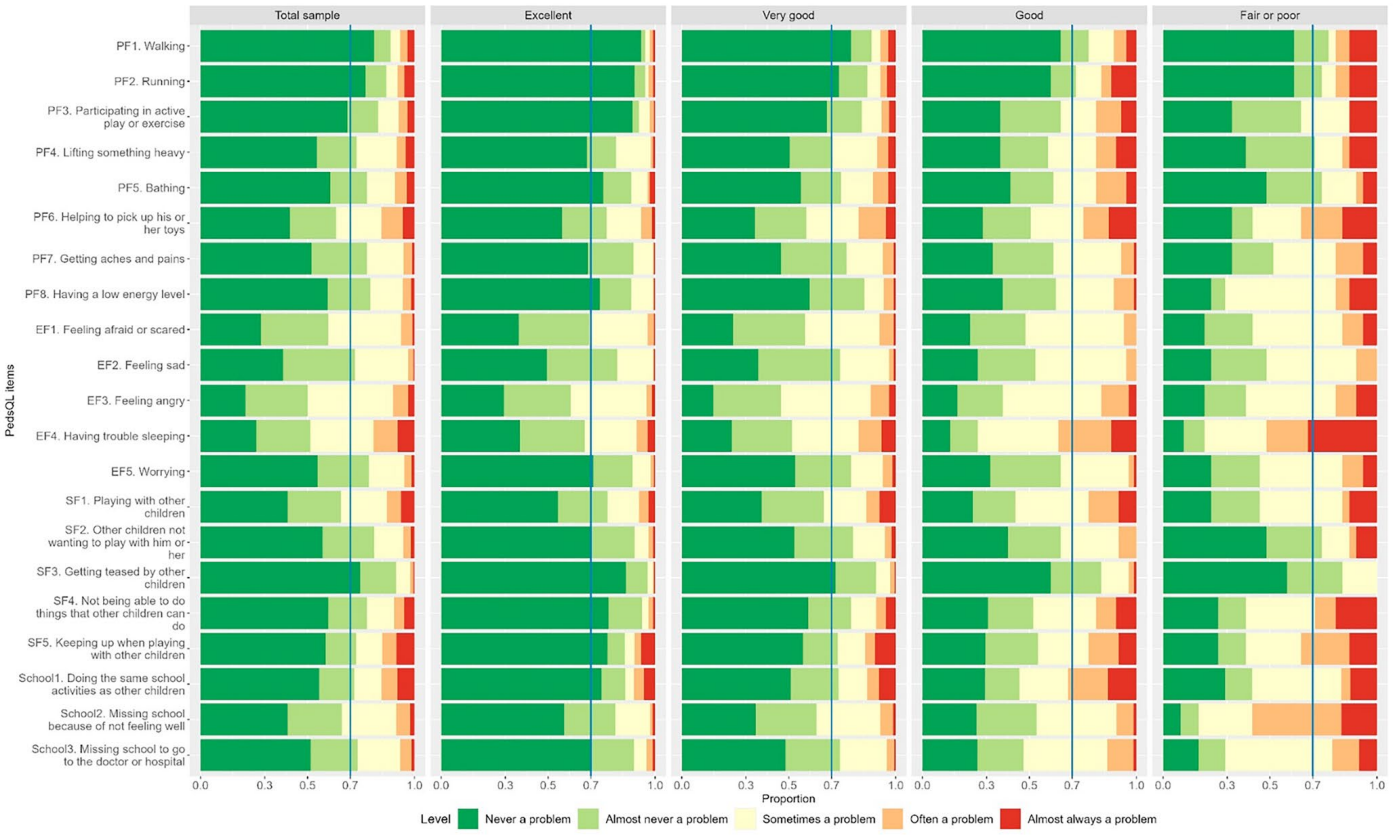
Response distribution of PedsQL items. Legend: Response distribution of PedsQL item for total sample and by general health groups. General health groups were identified based on parents/caregivers response to the general health question to describe their child’s general health today as ‘excellent’, ‘very good’, ‘good’, ‘fair’ or ‘poor’. Based on this question, children were categorized into four groups: (1) excellent, (2) very good, (3) good, (4) fair and poor. PedsQL, Paediatric Quality of Life Inventory. PF, physical functioning. EF, emotional functioning. SF, social functioning. School, school functioning

Figure 1 plots the response distribution of PedsQL items in the total sample and by general health groups. In the total sample, over 70% reported the best level for ‘Walking’, ‘Running’ and ‘Getting teased by other children’. ‘Feeling angry’ (79.0%), ‘Having trouble sleeping’ (73.9%), and ‘Feeling afraid or scared’ (71.8%) had the highest proportions of reporting problems. In the poor or fair health groups, items in emotional functioning and school functioning had higher proportions of reporting problems. The differences in response distributions across the general health status group were statistically significant at 5% level.

**Fig. 2:**
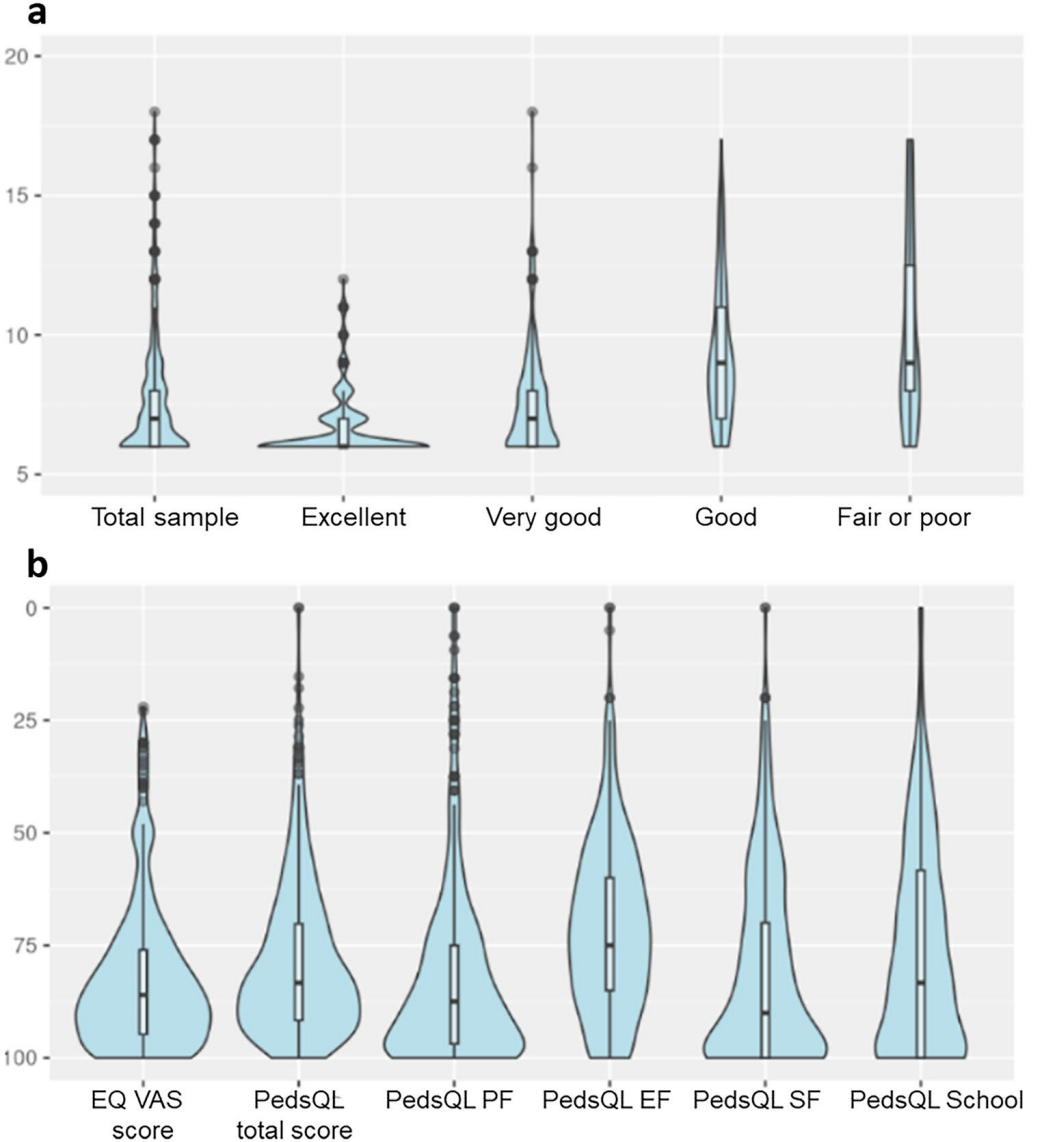
Distribution of EQ-TIPS level sum score, EQ VAS score and PedsQL total and domain scores (**a**) Distribution of EQ-TIPS level sum score for the total sample and by general health groups (**b**) Distribution of EQ VAS and PedsQL total score and domain scores for the total sample. Legend: Distribution of instrument summary scores for EQ-TIPS and PedsQL, plotted by violin plot. Figure 1a shows EQ-TIPS level sum scores which are on a 6–18 scale, the higher score represents worse health. Figure 1b shows EQ VAS score and PedsQL total score and domain scores which are on a 0-100 scale. Higher score represents better health. General health groups were identified based on parents/caregivers response to the general health question to describe their child’s general health today as ‘excellent’, ‘very good’, ‘good’, ‘fair’ or ‘poor’. Based on this question, children were categorized into four groups: (1) excellent, (2) very good, (3) good, (4) fair and poor. PedsQL, Paediatric Quality of Life Inventory. PF, physical functioning. EF, emotional functioning. SF, social functioning. School, school functioning

The proportion of total sample participants reporting ‘no problems’ for all dimensions of the EQ-TIPS (1111111) was 42.9%. Among children with proxy-reported fair or poor health, the proportion of reporting ‘no problems’ for all dimensions was 12.9% which indicates no ceiling effect. However, EQ-TIPS had a ceiling effect in the SHCN sample (25.8% of *n* = 159). In the total sample, only 1 child was reported to have ‘a lot of problems’ for all dimensions, therefore no floor effect was evident. Table A4 in ESM1 presents the number of unique EQ-TIPS health state profiles and the five most frequently reported profiles. 111111 and 111112 were the most two common health states reported in the total sample (42.9% and 8.63%), and in excellent (65.7% and 11.1%) and very good health status groups (37.8% and 9.7%). In the good health group, the three most frequently reported health states were 111111 (13.0%), 111222 (5.9%) and 112111 (5.9%). In the ‘poor or fair’ health group (*n* = 31), the top three health profiles were 111111 (12.9%), 332,333 (9.7%) and 111112 (6.5%). The mean (SD) EQ-TIPS LSS and EQ VAS scores were 7.6 (2.2) and 82.7 (16.1), and the mean PedsQL total score was 78.5 (17.2). There were 59 (11.6%) and 15 (2.9%) children having an EQ VAS score of 100 and PedsQL total score of 100 respectively. The distribution of EQ-TIPS LSS for the total sample and by general health groups is shown in Fig. 2 (a). The distribution of EQ VAS and PedsQL total score and domain scores for the total sample is shown in Fig. 2 (b) and by general health status groups in Figure A1 in ESM1.

### Known-group validity

Table 3 summarises the known-group validity with EQ-TIPS and PedsQL, both showing large significant effect sizes for differentiating children with or without SHCN, and differentiating those who had good, fair or poor health from those who had excellent health. EQ-TIPS was more sensitive than the PedsQL in distinguishing between those reporting the 3 specific conditions (sleep, atopy, and neurological conditions) with healthy children (free of any health conditions and had an EQ VAS score of at least 70 at baseline). As expected, there were no significant differences and relevant effect size in HRQOL between age 2 and age 3.

**Table 3:** Known group validity

	EQ-TIPS Level sum scores				PedsQL total scores			
Group	N	Mean (SD)	*p* value	effect size	N	Mean (SD)	*p* value	effect size
**General health status** (Excellent health group as Reference)								
Excellent	198	6.66(1.18)			198	86.65(12.64)		
very good	196	7.59(1.97)	< 0.001	0.57	196	77.56(16.44)	< 0.001	0.62
good	85	9.28(2.56)	< 0.001	1.32	85	67.4(17.31)	< 0.001	1.27
fair or poor	31	10.13(3.48)	< 0.001	1.34	31	62.17(18.69)	< 0.001	1.53
**Special health care needs**								
No	351	7.06(1.52)	< 0.001	0.83	351	82.83(13.69)	< 0.001	0.81
Yes	159	8.99(2.91)			159	68.81(20.25)		
**Sleep problems**								
No	144	6.56(0.94)	< 0.001	1.25	144	85.67(14.36)	< 0.001	1.15
Yes	75	9.31(2.97)			75	66.36(18.97)		
**Atopy**								
No	144	6.56(0.94)	< 0.001	0.81	144	85.67(14.36)	< 0.001	0.58
Yes	156	8.08(2.48)			156	76.62(16.82)		
**Neurological conditions**								
No	144	6.56(0.94)	< 0.001	1.87	144	85.67(14.36)	< 0.001	1.57
Yes	82	10.55(2.86)			82	60.57(17.41)		
**Age**								
2 years	263	7.71(2.41)	1	0.04	263	78.44(18.21)	1	0.00
3 years	247	7.62(2.06)			247	78.48(16.26)		

Cohen’s D effect size thresholds 0.2–0.49, 0.5–0.79 and > 0.8 denote small, medium and large effect size, respectively

The healthy group was free of any health condition and had an EQ VAS score of at least 70 at baseline

PedsQL Paediatric Quality of Life Inventory, SD standard deviation

### Convergent validity

As shown in Table 4, at the instrument level, EQ-TIPS LSS and PedsQL total score were strongly correlated. At the item level we found moderate to strong correlations in all pairs hypothesized to be correlated. In addition, we found strong correlations between EQ-TIPS Movement and PedsQL ‘Active play or exercise’, and between EQ-TIPS Social Interaction and Communication with PedsQL item ‘not able to do things that other children can do’.

**Table 4:** Correlations between PedsQL item and EQ-TIPS item

	EQ-TIPS							
	Movement	Play	Pain	Social interaction	Communication	Eating	EQ-TIPS LSS	
**PedsQL Physical functioning**								
walking	**-0.66**	-0.45	-0.28	-0.37	-0.38	-0.23	-0.44	
running	**-0.65**	-0.44	-0.22	-0.38	-0.39	-0.23	-0.44	
active play or exercise	-0.5	**-0.46**	-0.27	-0.45	-0.44	-0.27	-0.51	
lifting something heavy	-0.42	-0.33	-0.16	-0.27	-0.31	-0.24	-0.36	
bathing	-0.33	-0.35	-0.25	-0.31	-0.34	-0.29	-0.41	
helping to pick up toys	-0.31	-0.34	-0.24	-0.29	-0.34	-0.29	-0.41	
hurts or aches	-0.27	-0.26	**-0.41**	-0.18	-0.18	-0.25	-0.35	
low energy level	-0.3	-0.29	-0.31	-0.28	-0.25	-0.25	-0.36	
Sub domain score	-0.48	-0.43	-0.35	-0.4	-0.42	-0.36	-0.56	
**PedsQL Emotional functioning**								
afraid or scared	-0.15	-0.18	-0.23	-0.28	-0.17	-0.24	-0.29	
sad or blue	-0.16	-0.27	-0.33	-0.31	-0.23	-0.29	-0.36	
angry	-0.2	-0.24	-0.21	-0.27	-0.28	-0.29	-0.38	
trouble sleeping	-0.23	-0.28	-0.32	-0.25	-0.26	-0.34	-0.42	
worrying	-0.21	-0.26	-0.28	-0.33	-0.28	-0.28	-0.38	
Sub domain score	-0.25	-0.33	-0.37	-0.38	-0.33	-0.39	-0.50	
**PedsQL Social functioning**								
playing with other children	-0.27	**-0.34**	-0.24	**-0.49**	-0.4	-0.23	-0.45	
other kids not wanting to play with them	-0.24	-0.25	-0.16	-0.36	-0.34	-0.22	-0.37	
teased by other children	-0.1	-0.16	-0.09	-0.21	-0.22	-0.15	-0.26	
not able to do things that other children can do	-0.42	-0.43	-0.25	-0.54	-0.57	-0.36	-0.58	
keeping up when playing with other children	-0.35	**-0.39**	-0.27	-0.41	-0.43	-0.27	-0.46	
Sub domain score	-0.36	-0.41	-0.28	-0.53	-0.5	-0.32	-0.55	
**PedsQL School functioning**								
same school activities as peers	-0.38	-0.35	-0.24	-0.35	-0.43	-0.25	-0.44	
missing school because of not feeling well	-0.18	-0.23	-0.21	-0.2	-0.23	-0.2	-0.30	
missing school to go to doctor	-0.35	-0.35	-0.23	-0.33	-0.34	-0.23	-0.39	
Sub domain score	-0.36	-0.36	-0.29	-0.34	-0.39	-0.26	-0.45	
**PesQL total score**	-0.44	-0.45	-0.38	-0.5	-0.49	-0.41	-0.63	

Correlations were calculated using Spearman’s correlation. Correlations of 0.1–0.29 were considered weak, 0.3–0.49 moderate, and ≥ 0.5 strong (Cohen J 1992). Bold font indicates expected moderate or high correlations (r ≥ 0.3) based on highly similar items in line with published technical guide. PedsQL, Paediatric Quality of Life Inventory

### Test-retest reliability

Table 5 presents the test–retest results. EQ-TIPS had moderate to substantial agreement across the six items, and PedsQL had good to excellent agreement across the four domains. At the instrument level, both EQ-TIPS (ICC = 0.89) and PedsQL (ICC = 0.77) showed excellent agreement in the total sample.

**Table 5:** Test rest reliability

		Total sample (*N* = 173)
EQ-TIPS dimension		Weighted kappa (standard error)
Movement	kappa	0.58 (0.06)
Play	kappa	0.53 (0.07)
Pain	kappa	0.48(0.07)
Social Interaction	kappa	0.55 (0.06)
Communication	kappa	0.66 (0.06)
Eating	kappa	0.53 (0.06)
PedsQL domains		ICC (95% Confidence Interval)
Physical Functioning	ICC	0.71 (0.63–0.78)
Emotional Functioning	ICC	0.66 (0.57–0.74)
Social Functioning	ICC	0.76 (0.69–0.82)
School Functioning	ICC	0.67 (0.59–0.76)
Summary score		
EQ-TIPS LSS	ICC	0.89 (0.85–0.91)
PedsQL	ICC	0.77 (0.70–0.82)

ICC intraclass correlation coefficient; LSS: level sum score; PedsQL Paediatric Quality of Life Inventory

At dimension level, Weighted kappa coefficient was calculated. Coefficient values of 0.2, 0.21–0.40, 0.41–0.6, 0.61–0.80 and > 0.81 were indicative of poor, fair, moderate, substantial, and almost-perfect agreement, respectively

### Responsiveness

Table 6 presents the responsiveness of EQ-TIPS LSS and PedsQL total score for children whose caregivers reported a change in their general health status or main health condition during a period of four weeks. For children whose general health (*n* = 44) or main health condition (*n* = 33) was much better at the follow-up, the EQ-TIPS had a mean LSS change of -0.42 and − 0.73, which is in the direction expected. However, the changes were not statistically significant, and a small effect size was observed among those reporting improvements in the main health condition. PedsQL total scores had a mean change of 4.27 and 4.65 respectively. The PedsQL total score changes in the main health condition were statistically significant at 5% significance level, with a small effect size, which reached the MCID of 4.5 for proxy report [36].

**Table 6:** Responsiveness, by improvements in general health or in main health condition

Instruments	change in general health						change in main health condition					
	NϮ	Initial survey mean (SD)	Follow up survey mean (SD)	Mean difference (SD)	*p* value	SRM	*N*	Initial survey mean (SD)	Follow up survey mean (SD)	Mean difference (SD)	*p* value	SRM
Change in health - ‘much better’												
EQ-TIPS	43	7.67(2.77)	7.26(1.9)	-0.42(2.33)	0.25	-0.18	33	7.48(2.55)	6.76(1.2)	-0.73(2.44)	0.1	-0.3
PedsQL	43	78.61(20.09)	82.88(17.23)	4.27(14.75)	0.06	0.29	33	79.54(18.71)	84.19(16.33)	4.65(13.1)	0.05	0.35

PedsQL Paediatric Quality of Life Inventory, SD standard deviation, SRM Standardised response mean

Follow-up survey was 4 weeks post initial survey. SRM thresholds of 0.2–0.49, 0.5–0.79 and > 0.8 denote small, moderate, and large effect sizes, respectively

Ϯ sample size (criterion approach) *n* ≥ 50 in smallest group very good, n-30-50 adequate, *n* < 30 doubtful

### Subgroup analysis

Response distributions, known-group validity, convergent validity, test-retest reliability results for children aged 2 years and for children aged 3 years were fairly similar to the main results (ESM 2). There were stronger correlations between EQ-TIPS and PedsQL domains in children aged 2 years than those aged 3. EQ-TIPS LSS showed excellent test-retest reliability in both ages, PedsQL total scores showed excellent reliability in those age 2 with good reliability in age 3.

### Summary of psychometric performance

Table 7 summarises the performance of EQ-TIPS and PedsQL. EQ-TIPS showed ceiling effects in children with SHCNs. While both the EQ-TIPs and the PedsQL showed evidence of validity for known groups and test-retest reliability, the EQ-TIPS showed larger effect sizes in known-group validity and stronger correlation coefficients for test-retest reliability. PedsQL was more sensitive in detecting general health improvements.

**Table 7:** Summary of instrument psychometric performance

Instrument	Response distribution (no Ceiling effect) ^a^	Known group validity ^b^	Convergent validity ^c^	Test–retest reliability ^d^	Responsiveness ^e^
PedsQL	✔	✔	✔	✔	✔for improved health
EQ-TIPS	✗	✔	✔	✔	✗ for improved health

✓ Evidence of significant performance; ✗ No evidence of significant performance;? Inconclusive evidence

a ✓ Less than 15% of participants with a special healthcare need reported the lowest severity or frequency level (i.e., ‘no problems’) across all items

b ✓ Effect sizes ≥ 0.8

c ✓ Items at least moderately correlated (Spearman’s correlation ≥ 0.3) where items/instrument correlations where hypothesised

d ✓ ICC (good, or excellent) ≥ 0.6

e ✓ Mean difference with a p value of < 0.05

## Discussion

This study generated new evidence on the feasibility, reliability and validity of EQ-TIPS compared to PedsQL in children aged 2 and 3 years in Australia in the general population and across a range of health conditions. The EQ-TIPS, being a newer instrument, has less existing validity testing. Importantly, this study demonstrated both EQ-TIPS and PedsQL are feasible as a proxy report instrument to measure HRQOL of young children. EQ-TIPS took less time and was reported to be easier to complete compared to PedsQL. Both instruments showed comparable psychometric performance.

The EQ-TIPS demonstrated ceiling effects in the proxy-reported SHCN sample (25.8%), but not in children with poor or fair health (12.9%). One study found that the ceiling effects of EQ-5D-Y-3L adapted version for 2–4 years old in P-MIC was 22% and 11.5% for the SHCN and poor/fair health sample respectively. Children with SHCN were identified via a validated screening questionnaire whereas poor or fair health was a narrower classification based on parent-report of child health ‘today’. Table A5 in the ESM1 demonstrates that two measures captured different but overlapping constructs of health reported differently by parents. No ceiling effects were observed in PedsQL, which is consistent with literature and is expected [38] as higher ceiling effects are commonly observed for PROMs with fewer dimensions and fewer response levels, and using a shorter recall period [39, 40]. Studies from South Africa and China also found ceiling effects in health condition samples (between 28% and 41%) among 2-4-year-olds [15, 18, 20]. In another study using P-MIC data that compared the performance of the adapted EQ-5D-Y-3L and EQ-5D-5L in 2–4 years, the authors found ceiling effects in EQ-5D-Y-3L but not EQ-5D-Y-5L among children with SHCNs [41]. Unlike other EuroQol instruments, EQ-TIPS currently has one version with three response levels. The ceiling effects may be reduced when using more response levels [39, 42].

At the EQ-TIPS item level, eating had the highest proportion of reported problems in the full sample, followed by communication and social interaction. A similar pattern was observed in the validation study of EQ-TIPS in South Africa [16]. This is anticipated as eating or feeding problems are common in children and occur in 20-50% of healthy children [43–45]. In our sample, children who with proxy-reported fair or poor health status also had more problems (and higher proportion of ‘level 3’ problems) in eating than other dimensions. The study from China on young children with COVID-19 showed similar findings, but studies in South Africa found children aged 2–4 years across different health conditions had more problems in movement, play or communication [15, 18]. One explanation may be that the health conditions included in these studies differ thus impact health differently.

EQ-TIPS LSS showed stronger test-retest agreement compared to PedsQL total score in the total sample and in ages 2 and age 3 years. This may be explained by the general lability in this age group over the 4-week period between baseline and follow-up, and that PedsQL uses a longer recall period which may capture more variation. An alternative explanation is that PedsQL also detect problems that are common across childhood which are not necessarily affected only by ill-health. For example, even among children with excellent health, there were high reporting of problems in helping to pick up toys, playing with other children, missing school because of not feeling well. At the item/domain level, the EQ-TIPS communication item showed substantial agreement while other items moderate; PedsQL domain scores showed good to excellent agreement, with the Emotional Functioning domain having lower ICC compared to the other domains. A recent study examining validity of PedsQL in children with suspected genetic conditions and found relatively poor reliability for this domain [46]. For EQ-TIPS, one previous study has examined its test-retest reliability among 23 children from the general population and there was little variance in the dimensions [16]. Studies have found fair to excellent reliability on EQ-5D-Y-3L, EQ-5D-Y-5L LSS [41, 42, 47], and poor to substantial agreement at the item level, particularly, poor or fair agreement for pain/discomfort and worried, sad or unhappy in younger age groups [41, 42, 48]. Compared to the less favourable reliability with EQ-5D-Y-3L and EQ-5D-Y-L in young children, the EQ-TIPS and PedsQL showed good test-retest reliability and the reasons may be that both were designed specifically for young children and the wording of dimensions may be easier for proxy report. For example, in EQ-TIPS, the pain dimension is described with examples “painful behaviour includes grimace, restless movement, inconsolable cry”, whereas in EQ-5D-Y-3L and EQ-5D-Y-5L instruments no examples are given in the pain/discomfort dimension headings.

Both PedsQL and EQ-TIPS showed good known-group validity and were able to distinguish by general health status, children’s special health needs, and health conditions. Previous studies showed EQ-TIPS and PedsQL was able to discriminate between levels of severity of health conditions [15, 16, 18]. A recent study found PedsQL was able to differentiate children with colorectal conditions aged 2–4 years in Australia [49]. We add to the evidence base that EQ-TIPS showed good discriminate validity in a range of health conditions and was more sensitive to distinguish these conditions compared to PedsQL.

We found moderate to strong correlations in all pairs hypothesized to be correlated. The correlations were stronger in children aged 2 years than those aged 3, which may reflect the developmental progression with more interdependence of developmental skills in younger children [50]. We also found the EQ-TIPS social interaction and communication and PedsQL social functioning items were moderately-to-strongly correlated. In the EQ-TIPS development work, communication after a child reaches age 24 months refers more to the interaction with other children which is also emphasised in all items in the social functioning domain in PedsQL. EQ-TIPS eating and pain were correlated with fewer PedsQL items, which is consistent with previous findings [15]. This is likely because PedsQL does not directly measure eating and only included one item on pain. Given the relatively high prevalence of eating problems in young children, it may be important to consider this aspect when measuring HRQOL. The PedsQL emotional functioning domain has less proportion of items correlated with EQ-TIPS dimensions and LSS compared with other PedsQL domains. This finding is consistent with previous studies comparing PedsQL with EQ-TIPS or other generic measures (e.g. EQ VAS) in children with health conditions [46, 51]. In EQ-TIPS, emotional aspects were not explicitly included as a dimension, although it was expected that these aspects might be captured indirectly through social interaction and communication [9, 18, 19]. The PedsQL in contrast has dedicated items for afraid, sad, angry, and worried. Our finding suggests more work may be needed to explore the representation of mental health and emotional functioning in EQ-TIPS. This may also be relevant to considerations around the continuity in measuring HRQOL in young children and over the life span using the EuroQol instruments, as both EQ-5D adult and youth instruments have an explicit dimension on mental health.

In our responsiveness analysis, changes in EQ-TIPS and PedsQL scores were in the direction expected. We only observed non-significant trivial to small effect sizes using EQ-TIPS in children with improved health. For PedsQL, we observed statistically significant and small effect sizes in children with caregiver-reported improvement in their health condition, and the difference in mean scores reached the MCID of 4.5 for proxy report [36]. To date, only one study examined responsiveness of EQ-TIPS, which found strong responsiveness evidence of EQ-TIPS LSS to health improvements among paediatric patients being affected by COVID-19 based on clinical indicators and parent-reported general health improvement, 1–3 weeks after baseline. In our study, we identified health improvements based on proxy-reported improvements perceived by caregivers, which is a weaker indicator for changes in health compared to an objective or clinical indicator or using pre-post assessment in the context of an intervention.

Overall, our results represent the first evidence on the comparison of the validity and reliability of the EQ-TIPS and PedsQL in children aged 2 and 3 years in Australia. Our study benefited from using a relatively large sample and the inclusion of a wide range of health conditions. However, there are several caveats. First, EQ-TIPS was designed for children aged 1–36 months [15, 18]. Our EQ-TIPS data from the P-MIC study only included children aged 2–3 years. Future research should explore the validity of EQ-TIPS in infants (1–24 months). Second, although the P-MIC study included a wide range of conditions, this information was provided via parent report rather than hospital records or clinician assessments. The sample size for each condition was relatively small and the number of children with severe health conditions was limited. The sample size to test responsiveness for improved health was small, and we were not able to explore responsiveness to worsening health. However, it might be noted that omitting worsened health is not a major concern as it was relatively rare among children, especially within a period of 4 weeks. The responsiveness analysis also has limitations as it was not conducted in the context of interventions or treatments. A sample of children recruited with treatments and clinical measures available would provide more information about instrument responsiveness. Third, we used LSS for EQ-TIPS as a crude summary score in the absence of HRQoL values. However, different health profiles may have the same LSS [52]. Using LSS assumes equal weight across the dimensions. Nevertheless, considering the current lack of preference-weighted scores, the current analysis provides an indication of how the instruments perform at an aggregated level. Further research is required to examine the performance of EQ-TIPS applying the HRQoL values when it becomes available [53].

## Conclusion

The EQ-TIPS and the PedsQL demonstrated good psychometric performance in children aged 2 and 3 years in Australia. Whilst neither instrument currently has the ability to be scored as HRQoL values for incorporation in economic evaluation, the EQ-TIPS has specifically been designed for this purpose [53]. Two HSCS for PedsQL have now been developed for eliciting preference for use in children 2 years and above [5, 6]. Regardless, our results have implications for instrument developers and potential users. There is relatively little prior evidence regarding psychometric performance of HRQoL instruments for young children and the current study comparing PedsQL and EQ-TIPS will provide guidance to those wishing to measure HRQoL of young children. Both the PedsQL and EQ-TIPS are valid options with small differences in performance depending on the users’ needs and context. Compared with PedsQL, EQ-TIPS has fewer items and was found to be easier to complete across all general health status groups, which may be more suitable for use in large-scale child health population surveys. EQ-TIPS also showed stronger effect sizes in differentiating children with health conditions and was more sensitive to issues around pain and eating whereas PedsQL showed statistical significance and changes greater than the MCID in detecting improvements in health and was more sensitive to emotional aspects. Overall, the performance was similar in children ages 2 and 3 years. This new evidence can inform consideration of the choice of instrument.

## Electronic supplementary material

Below is the link to the electronic supplementary material.

Supplementary Material

## Data Availability

The datasets generated during and/or analysed during the current study are available from the corresponding author on reasonable request.
