# Supplementary material for: Comparison of the psychometric performance of experimental EuroQol Toddler and Infant Populations Instrument (EQ-TIPS) and Pediatric Quality of Life Inventory™ (PedsQL) in young children
Source: Eur J Health Econ. 2025 Jul 30;27(2):289–304. doi: 10.1007/s10198-025-01804-4 (PMC13046681; doi:10.1007/s10198-025-01804-4)
Supplement: Supplementary file 1 — Supplementary Material [file 10198_2025_1804_MOESM1_ESM.docx]

**Supplementary Material 1**

| Table A1. Sample characteristics at baseline, by general health status group | | | | |
| --- | --- | --- | --- | --- |
|  | Excellent | Very good | Good | Fair or poor |
|  | (N=198) | (N=196) | (N=85) | (N=31) |
| **Sample source (Sample 1, 2, 3)** |  |  |  |  |
| Sample 1 (children with or without health conditions recruited via a large tertiary paediatric hospital) | 71(35.9%) | 82(41.8%) | 48(56.5%) | 22(71%) |
| Sample 2 (general population recruited via an online panel) | 119(60.1%) | 100(51%) | 25(29.4%) | 4(12.9%) |
| Sample 3 (specific conditions recruited via an online panel – for EQ-TIPS sample only includes sleep problems) | 8(4%) | 14(7.1%) | 12(14.1%) | 5(16.1%) |
| **Child gender** |  |  |  |  |
| Male | 97(49%) | 102(52%) | 50(58.8%) | 17(54.8%) |
| Female | 99(50%) | 94(48%) | 34(40%) | 14(45.2%) |
| Other | 2(1%) | 0(0%) | 1(1.2%) | 0(0%) |
| **Child age (years)** |  |  |  |  |
| 2 | 107(54%) | 107(54.6%) | 35(41.2%) | 14(45.2%) |
| 3 | 91(46%) | 89(45.4%) | 50(58.8%) | 17(54.8%) |
| **Aboriginal or Torress Strait Island origin** |  |  |  |  |
| Yes | 11(5.6%) | 9(4.6%) | 5(5.9%) | 1(3.2%) |
| No | 186(93.9%) | 187(95.4%) | 80(94.1%) | 30(96.8%) |
| Prefer not to say | 1(0.5%) | 0(0%) | 0(0%) | 0(0%) |
| **Child speaks language other than English at home** |  |  |  |  |
| No, the child speaks English at home | 164(82.8%) | 161(82.1%) | 71(83.5%) | 26(83.9%) |
| **Caregiver education-bachelor’s degree or above** |  |  |  |  |
| Yes | 101(51%) | 109(55.6%) | 36(42.4%) | 16(51.6%) |
| **Single parent household** |  |  |  |  |
| Yes | 25(12.8%) | 27(13.8%) | 15(18.1%) | 5(16.7%) |
| **Household weekly income before tax (AUD)** |  |  |  |  |
| Less than $500 per week ($25,999 or less per year) | 8(4.1%) | 8(4.3%) | 2(2.5%) | 3(9.7%) |
| $500-$999 per week ($26,000-$51,999 per year) | 33(16.8%) | 31(16.5%) | 18(22.2%) | 5(16.1%) |
| $1,000-$1,999 per week ($52,000-$103,9799 per year) | 74(37.6%) | 68(36.2%) | 32(39.5%) | 14(45.2%) |
| $2,000 or more per week ($104,000 or more per year) | 82(41.6%) | 81(43.1%) | 29(35.8%) | 9(29%) |
| **Remoteness** |  |  |  |  |
| Major cities | 147(74.2%) | 157(80.1%) | 63(74.1%) | 21(67.7%) |
| Inner regional | 40(20.2%) | 27(13.8%) | 16(18.8%) | 8(25.8%) |
| Outer regional | 8(4%) | 10(5.1%) | 6(7.1%) | 2(6.5%) |
| Remote/very remote | 3(1.5%) | 2(1%) | 0(0%) | 0(0%) |
| **Completed 4-week follow up survey** |  |  |  |  |
| Yes | 102(59.3%) | 111(63.4%) | 54(68.4%) | 19(61.3%) |
| **Health conditions with prevalence >5%*** |  |  |  |  |
| Constipation | 26(13.1%) | 39(19.9%) | 25(29.4%) | 6(19.4%) |
| Developmental delay | 12(6.1%) | 22(11.2%) | 11(12.9%) | 6(19.4%) |
| Eczema | 8(4%) | 12(6.1%) | 6(7.1%) | 1(3.2%) |
| Food or digestive allergies | 9(4.6%) | 31(15.8%) | 15(17.7%) | 6(19.4%) |
| Hay fever | 4(2%) | 7(3.6%) | 13(15.3%) | 6(19.4%) |
| Asthma | 9(4.6%) | 27(13.8%) | 28(32.9%) | 11(35.5%) |
| Behavioural, cognitive & emotional problems | 4(2%) | 7(3.6%) | 13(15.3%) | 6(19.4%) |
| Sleep problems | 9(4.6%) | 27(13.8%) | 28(32.9%) | 11(35.5%) |
| **Healthy reference group (i.e., without any ongoing conditions and having a EQ VAS score of at least 70)** |  |  |  |  |
| Yes | 86(100%) | 54(100%) | 3(100%) | 1(100%) |
| **Change in general health** |  |  |  |  |
| much better | 19(18.6%) | 13(11.7%) | 10(18.5%) | 1(5.3%) |
| somewhat better | 16(15.7%) | 30(27%) | 10(18.5%) | 4(21.1%) |
| about the same | 67(65.7%) | 67(60.4%) | 29(53.7%) | 10(52.6%) |
| somewhat or much worse | 0(0%) | 1(0.9%) | 5(9.3%) | 4(21.1%) |
| **Change in main health conditions** |  |  |  |  |
| much better | 14(24.1%) | 11(13.9%) | 8(15.4%) | 0(0%) |
| somewhat better | 4(6.9%) | 20(25.3%) | 12(23.1%) | 3(15.8%) |
| about the same | 40(69%) | 47(59.5%) | 29(55.8%) | 12(63.2%) |
| somewhat or much worse | 0(0%) | 1(1.3%) | 3(5.8%) | 4(21.1%) |
| All information were collected by parents/caregiver proxy report. | | | | |
| *The survey asked about ongoing conditions from a list of 43. We report all those with prevalence of >5% for children aged 2/3 years included in this analysis. | | | | |

| Table A2 Time to completion (seconds) | | | | | | | | | | |
| --- | --- | --- | --- | --- | --- | --- | --- | --- | --- | --- |
|  | EQ-TIPS | | | | | PedsQL | | | | |
|  | Total  Sample | Excellent | Very  Good | Good | Fair or poor | Total  Sample | Excellent | Very  Good | Good | Fair or poor |
| Mean | 48.9 | 47.5 | 50.9 | 46.2 | 52.2 | 152.7 | 192.5 | 135.3 | 108.4 | 120.3 |
| SD | 156.4 | 155.1 | 196.4 | 42 | 39.1 | 731.4 | 1110.2 | 353.8 | 52.4 | 60 |
| Median | 27.1 | 23.9 | 27.7 | 36.6 | 37.2 | 86.2 | 75.2 | 87.4 | 95.9 | 105.4 |
| 25% quantile | 19.9 | 17.8 | 20.1 | 25 | 28.5 | 64.3 | 60.5 | 64.8 | 70.8 | 80.9 |
| 75% quantile | 39.9 | 31.8 | 38.9 | 49.9 | 60.1 | 120 | 102.9 | 127 | 130.3 | 124.6 |
| General health groups were identified based on parents/caregivers response to the general health question to describe their child’s general health today as ‘excellent’, ‘very good’, ‘good’, ‘fair’ or ‘poor’. Based on this question, children were categorized into four groups: 1) excellent, 2) very good, 3) good, 4) fair and poor.  PedsQL Paediatric Quality of Life Inventory, SD standard deviation | | | | | | | | | | |

| Table A3 Difficulty to complete the instrument (%) | | | | | | | | | | |
| --- | --- | --- | --- | --- | --- | --- | --- | --- | --- | --- |
|  | EQ-TIPS | | | | | PedsQL | | | | |
| Difficulty to complete | Total  Sample | Excellent | Very  Good | Good | Fair or poor | Total  Sample | Excellent | Very  Good | Good | Fair or poor |
| 1 very difficult | 1 | 1 | 1 | 1 | 0 | 1 | 0 | 1 | 2 | 3 |
| 2 somewhat difficult | 2 | 2 | 1 | 4 | 3 | 5 | 5 | 4 | 7 | 3 |
| 3 neither difficult or easy | 10 | 4 | 8 | 24 | 16 | 14 | 6 | 15 | 28 | 26 |
| 4 somewhat easy | 21 | 16 | 23 | 29 | 23 | 25 | 25 | 27 | 22 | 23 |
| 5 very easy | 66 | 78 | 67 | 42 | 58 | 55 | 65 | 53 | 40 | 45 |
| General health groups were identified based on parents/caregivers response to the general health question to describe their child’s general health today as ‘excellent’, ‘very good’, ‘good’, ‘fair’ or ‘poor’. Based on this question, children were categorized into four groups: 1) excellent, 2) very good, 3) good, 4) fair and poor.  PedsQL Paediatric Quality of Life Inventory, SD standard deviation | | | | | | | | | | |

| Table A4 Top 5 most frequently reported EQ-TIPS health state profile | | | | | | | | | | |
| --- | --- | --- | --- | --- | --- | --- | --- | --- | --- | --- |
|  | Total sample | % | Excellent | % | Very good | % | Good | % | Fair or poor | % |
| No of unique profiles | 97 |  | 32 |  | 46 |  | 53 |  | 24 |  |
| Top 5 profile | | | | | | | | | | |
| 1 | 111111 | 42.94 | 111111 | 65.66 | 111111 | 37.76 | 111111 | 12.94 | 111111 | 12.9 |
| 2 | 111112 | 8.63 | 111112 | 11.11 | 111112 | 9.69 | 111222 | 5.88 | 332333 | 9.68 |
| 3 | 112111 | 3.33 | 111121 | 3.03 | 111122 | 4.59 | 112111 | 5.88 | 111112 | 6.45 |
| 4 | 111121 | 3.14 | 111211 | 2.53 | 111222 | 4.59 | 111121 | 3.53 | 112112 | 6.45 |
| 5 | 111222 | 2.94 | 111221 | 1.52 | 112111 | 4.59 | 111122 | 3.53 | 112113 | 6.45 |
| General health groups were identified based on parents/caregivers response to the general health question to describe their child’s general health today as ‘excellent’, ‘very good’, ‘good’, ‘fair’ or ‘poor’. Based on this question, children were categorized into four groups: 1) excellent, 2) very good, 3) good, 4) fair and poor.  EQ-TIPS has 729 theortically possible profiles | | | | | | | | | | |

| Table A5 Distribution of children with special health care needs by general health status | | | |
| --- | --- | --- | --- |
|  | Child has Special Health Care Needs | |  |
| Parent reported general health | No | Yes | Total |
| Excellent | 165(47.01%) | 33(20.75%) | 198(38.82%) |
| Very good | 141(40.17%) | 55(34.59%) | 196(38.43%) |
| Good | 36(10.26%) | 49(30.82%) | 85(16.67%) |
| Fair or poor | 9(2.56%) | 22(13.84%) | 31(6.08%) |
| Total | 351 | 159 | 510 |

Fig A1. Distribution of EQ VAS and PedsQL total score and domain scores for by general health groups


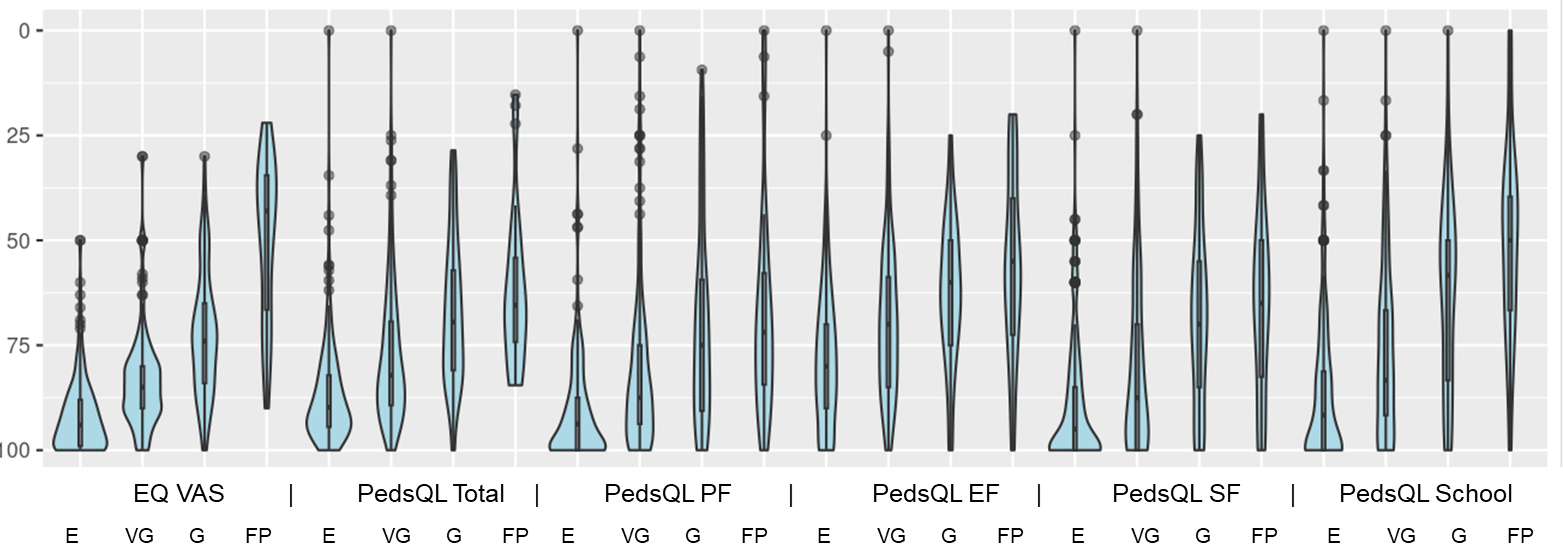


EQ VAS score and PedsQL total score and domain scores which are on a 0-100 scale. Higher score represents better health. General health groups were identified based on parents/caregivers response to the general health question to describe their child’s general health today as ‘excellent’, ‘very good’, ‘good’, ‘fair’ or ‘poor’. Based on this question, children were categorized into four groups: 1) excellent, 2) very good, 3) good, 4) fair and poor. PedsQL, Paediatric Quality of Life Inventory. PF, physical functioning. EF, emotional functioning. SF, social functioning. School, school functioning. E: Excellent. VG: Very good. G: Good. FP: Fair or poor.

**Supplementary Material 2: Subgroup analysis**

**Aged 2 years**

| Table B1 – Response distribution, in children aged 2 years | | | | | | |
| --- | --- | --- | --- | --- | --- | --- |
| Dimension | Total sample | General health groups | | | |  |
|  |  | Excellent | Very good | Good | Fair or poor | p-value (Fisher’s) |
| **Movement** |  |  |  |  |  | <0.001 |
| Level 1 | 220 (83.7%) | 102 (95%) | 89 (83%) | 23 (66%) | 6 (43%) |  |
| Level 2 | 34 (12.9%) | 4 (3.7%) | 16 (15%) | 10 (29%) | 4 (29%) |  |
| Level 3 | 9 (3.4%) | 1 (0.9%) | 2 (1.9%) | 2 (5.7%) | 4 (29%) |  |
| **Play** |  |  |  |  |  | <0.001 |
| Level 1 | 225 (85.6%) | 103 (96%) | 93 (87%) | 22 (63%) | 7 (50%) |  |
| Level 2 | 31 (11.8%) | 4 (3.7%) | 12 (11%) | 12 (34%) | 3 (21%) |  |
| Level 3 | 7 (2.7%) |  | 2 (1.9%) | 1 (2.9%) | 4 (29%) |  |
| **Pain** |  |  |  |  |  | <0.001 |
| Level 1 | 220 (83.7%) | 103 (96%) | 89 (83%) | 20 (57%) | 8 (57%) |  |
| Level 2 | 41 (15.6%) | 4 (3.7%) | 17 (16%) | 14 (40%) | 6 (43%) |  |
| Level 3 | 2 (0.8%) |  | 1 (0.9%) | 1 (2.9%) |  |  |
| **Social**  **interaction** |  |  |  |  |  | <0.001 |
| Level 1 | 190 (72.2%) | 93 (87%) | 79 (74%) | 15 (43%) | 3 (21%) |  |
| Level 2 | 57 (21.7%) | 12 (11%) | 24 (22%) | 15 (43%) | 6 (43%) |  |
| Level 3 | 16 (6.1%) | 2 (1.9%) | 4 (3.7%) | 5 (14%) | 5 (36%) |  |
| **Communication** |  |  |  |  |  | <0.001 |
| Level 1 | 182 (69.2%) | 93 (87%) | 72 (67%) | 13 (37%) | 4 (29%) |  |
| Level 2 | 53 (20.2%) | 10 (9.3%) | 25 (23%) | 14 (40%) | 4 (29%) |  |
| Level 3 | 28 (10.6%) | 4 (3.7%) | 10 (9.3%) | 8 (23%) | 6 (43%) |  |
| **Eating** |  |  |  |  |  | <0.001 |
| Level 1 | 172 (65.4%) | 92 (86%) | 64 (60%) | 12 (34%) | 4 (29%) |  |
| Level 2 | 73 (27.8%) | 15 (14%) | 37 (35%) | 18 (51%) | 3 (21%) |  |
| Level 3 | 18 (6.8%) |  | 6 (5.6%) | 5 (14%) | 7 (50%) |  |
| ceiling effect | 116 (44.1%) | 74 (69.2%) | 38 (35.5%) | 3 (8.6%) | 1 (7.1%) | <0.001 |
| LSS (mean, SD) | 7.71 ( 2.41 ) | 6.59 (1.17) | 7.69 (2.1) | 9.63 (2.6) | 11.57 (3.92) | <0.001 |
| EQ VAS (mean, SD) | 83.24 (16.24) | 92.43 (8.3) | 83.18(12.23) | 69.06 (15.9) | 49 (19.35) | <0.001 |
| Level 1: no problems, Level 2: some problems; Level 3: a lot of problems  General health groups were identified based on parents/caregivers response to the general health question to describe their child’s general health today as ‘excellent’, ‘very good’, ‘good’, ‘fair’ or ‘poor’. Based on this question, children were categorized into four groups: 1) excellent, 2) very good, 3) good, 4) fair and poor. | | | | | | |

| Table B2. Known-group validity in children aged 2 years | | | | | | | | | | |
| --- | --- | --- | --- | --- | --- | --- | --- | --- | --- | --- |
|  | EQ-TIPS Level sum scores | | | | | PedsQL total scores | | | | |
| Groups | N | Mean | SD | p value | effect size | N | Mean | SD | p value | effect size |
| **General health** | | | | | | | | | | |
| Excellent | 107 | 6.59 | 1.17 | <0.001 | 0.31 | 107 | 87.33 | 13.06 | <0.001 | 0.26 |
| very good | 107 | 7.69 | 2.1 |  |  | 107 | 76.94 | 16.73 |  |  |
| good | 35 | 9.63 | 2.6 |  |  | 35 | 64.96 | 16.86 |  |  |
| fair or poor | 14 | 11.57 | 3.92 |  |  | 14 | 55.56 | 23.29 |  |  |
| **Special health care needs** | | | | | | | | | | |
| No | 170 | 7 | 1.69 | <0.001 | 0.9 | 170 | 83.49 | 13.53 | <0.001 | 0.85 |
| Yes | 93 | 9 | 2.94 |  |  | 93 | 69.2 | 21.78 |  |  |
| **Chronic health condition** | | | | | | | | | | |
| No | 158 | 6.87 | 1.51 | <0.001 | 0.97 | 158 | 83.86 | 13.52 | <0.001 | 0.8 |
| Yes | 105 | 8.97 | 2.9 |  |  | 105 | 70.27 | 21.15 |  |  |
| **Eczema** |  |  |  |  |  |  |  |  |  |  |
| No | 76 | 6.45 | 0.76 | <0.001 | 1 | 76 | 86.59 | 12.66 | <0.001 | 0.63 |
| Yes | 54 | 8.33 | 2.78 |  |  | 54 | 76.84 | 18.63 |  |  |
| **Sleep problems** |  |  |  |  |  |  |  |  |  |  |
| No | 76 | 6.45 | 0.76 | <0.001 | 2.55 | 76 | 86.59 | 12.66 | <0.001 | 1.79 |
| Yes | 24 | 11.12 | 3.53 |  |  | 24 | 58.4 | 23.12 |  |  |
| **Asthma** |  |  |  |  |  |  |  |  |  |  |
| No | 76 | 6.45 | 0.76 | <0.001 | 1.37 | 76 | 86.59 | 12.66 | <0.001 | 0.89 |
| Yes | 30 | 8.4 | 2.42 |  |  | 30 | 74.6 | 15.56 |  |  |
| **Developmental delay** |  |  |  |  |  |  |  |  |  |  |
| No | 76 | 6.45 | 0.76 | <0.001 | 3.28 | 76 | 86.59 | 12.66 | <0.001 | 2.1 |
| Yes | 33 | 11.45 | 2.54 |  |  | 33 | 55.45 | 19.02 |  |  |
| **Food allergies** |  |  |  |  |  |  |  |  |  |  |
| No | 76 | 6.45 | 0.76 | <0.001 | 0.97 | 76 | 86.59 | 12.66 | <0.001 | 0.51 |
| Yes | 31 | 7.84 | 2.41 |  |  | 31 | 79.73 | 14.86 |  |  |
| **Atopy** |  |  |  |  |  |  |  |  |  |  |
| No | 76 | 6.45 | 0.76 | <0.001 | 0.83 | 76 | 86.59 | 12.66 | <0.001 | 0.62 |
| Yes | 86 | 8.05 | 2.53 |  |  | 86 | 77.3 | 16.85 |  |  |
| **Brain related Disorders** |  |  |  |  |  |  |  |  |  |  |
| No | 76 | 6.45 | 0.76 | <0.001 | 2.61 | 76 | 86.59 | 12.66 | <0.001 | 2.01 |
| Yes | 42 | 11.14 | 2.85 |  |  | 42 | 56.43 | 18.54 |  |  |
| Cohen’s D effect size thresholds 0.2–0.49, 0.5–0.79 and >0.8 denote small, medium and large effect size, respectively.  The healthy group was free of any chronic condition and had an EQ VAS score of at least 70 at baseline.  PedsQL Paediatric Quality of Life Inventory, SD standard deviation | | | | | | | | | | |

| Table B3 Test-retest reliability analysis among children aged 2 years (N=90) | | |
| --- | --- | --- |
| EQ-TIPS dimension |  | Weighted kappa (standard error) |
| Movement | kappa | 0.57(0.09) |
| Play | kappa | 0.58(0.09) |
| Pain | kappa | 0.41(0.10) |
| Social Interaction | kappa | 0.56(0.09) |
| Communication | kappa | 0.64(0.08) |
| Eating | kappa | 0.57(0.08) |
| PedsQL domains |  | ICC (95% Confidence Interval) |
| Physical Functioning | ICC | 0.76(0.65 -0.83) |
| Emotional Functioning | ICC | 0.71(0.60-0.80) |
| Social Functioning | ICC | 0.76(0.66 – 0.84) |
| School Functioning | ICC | 0.68(0.56 – 0.79) |
| Summary score |  |  |
| EQ-TIPS LSS | ICC | 0.90 (0.86 -0.94) |
| PedsQL | ICC | 0.81(0.73-0.87) |
| ICC intraclass correlation coefficient; LSS: level sum score; PedsQL Paediatric Quality of Life Inventory.  At dimension level, Weighted kappa coefficient was calculated. Coefficient values of 0.2, 0.21–0.40, 0.41–0.6, 0.61–0.80 and >0.81 were indicative of poor, fair, moderate, substantial, and almost-perfect agreement, respectively.  ICC is based on comparison of EQ-TIPS LSS, PedsQL domain and total score at initial and follow-up survey for participants who completed follow-up 4 weeks after initial survey and reported no change in health; ICC thresholds of <0.4, 0.4–0.59, 0.60–0.74 and > 0.75 were indicative of poor, fair, good, and excellent reliability, respectively | | |

| Table B4. Correlations between PedsQL item and EQ-TIPS item, among children aged 2 years old | | | | | | | |  |
| --- | --- | --- | --- | --- | --- | --- | --- | --- |
|  | EQ-TIPS | | | | | | |  |
|  | movement | play | pain | social interaction | communication | eating | EQ-TIPS LSS |  |
| **PedsQL Physical functioning** |  |  |  |  |  |  |  |  |
| walking | -0.71 | -0.54 | -0.28 | -0.42 | -0.43 | -0.36 | -0.54 |  |
| running | -0.73 | -0.54 | -0.27 | -0.42 | -0.44 | -0.39 | -0.55 |  |
| active play or exercise | -0.59 | -0.54 | -0.29 | -0.49 | -0.49 | -0.35 | -0.56 |  |
| lifting something heavy | -0.51 | -0.33 | -0.19 | -0.29 | -0.34 | -0.32 | -0.42 |  |
| bathing | -0.36 | -0.36 | -0.23 | -0.34 | -0.34 | -0.29 | -0.37 |  |
| helping to pick up toys | -0.37 | -0.35 | -0.25 | -0.26 | -0.31 | -0.26 | -0.36 |  |
| hurts or aches | -0.33 | -0.26 | -0.36 | -0.17 | -0.17 | -0.23 | -0.28 |  |
| low energy level | -0.33 | -0.33 | -0.36 | -0.31 | -0.24 | -0.29 | -0.35 |  |
| Sub domain score | -0.57 | -0.46 | -0.31 | -0.40 | -0.43 | -0.38 | -0.54 |  |
| **PedsQL Emotional functioning** |  |  |  |  |  |  |  |  |
| afraid or scared | -0.19 | -0.23 | -0.28 | -0.35 | -0.18 | -0.27 | -0.32 |  |
| sad or blue | -0.20 | -0.33 | -0.38 | -0.34 | -0.26 | -0.32 | -0.36 |  |
| angry | -0.30 | -0.31 | -0.22 | -0.26 | -0.33 | -0.24 | -0.37 |  |
| trouble sleeping | -0.31 | -0.35 | -0.33 | -0.27 | -0.30 | -0.38 | -0.45 |  |
| worrying | -0.27 | -0.34 | -0.23 | -0.39 | -0.30 | -0.34 | -0.38 |  |
| Sub domain score | -0.33 | -0.42 | -0.39 | -0.42 | -0.36 | -0.41 | -0.51 |  |
| **PedsQL Social functioning** |  |  |  |  |  |  |  |  |
| playing with other children | -0.33 | -0.38 | -0.25 | -0.52 | -0.43 | -0.29 | -0.50 |  |
| other kids not wanting to play with them | -0.28 | -0.27 | -0.16 | -0.41 | -0.41 | -0.22 | -0.38 |  |
| teased by other children | -0.19 | -0.14 | -0.15 | -0.21 | -0.22 | -0.16 | -0.25 |  |
| not able to do things that other children can do | -0.50 | -0.50 | -0.21 | -0.57 | -0.65 | -0.40 | -0.63 |  |
| keeping up when playing with other children | -0.44 | -0.46 | -0.25 | -0.48 | -0.53 | -0.33 | -0.52 |  |
| Sub domain score | -0.43 | -0.46 | -0.24 | -0.58 | -0.56 | -0.36 | -0.59 |  |
| **PedsQL School functioning** |  |  |  |  |  |  |  |  |
| same school activities as peers | -0.45 | -0.43 | -0.15 | -0.40 | -0.51 | -0.27 | -0.48 |  |
| missing school because of not feeling well | -0.27 | -0.31 | -0.19 | -0.25 | -0.26 | -0.24 | -0.36 |  |
| missing school to go to doctor | -0.42 | -0.42 | -0.28 | -0.40 | -0.35 | -0.27 | -0.47 |  |
| Sub domain score | -0.43 | -0.44 | -0.24 | -0.40 | -0.43 | -0.29 | -0.50 |  |
| **PesQL total score** | -0.53 | -0.50 | -0.34 | -0.52 | -0.52 | -0.44 | -0.64 |  |
| Correlations were calculated using Spearman’s correlation. Correlations of 0.1–0.29 were considered weak, 0.3–0.49 moderate, and ≥ 0.5 strong (Cohen J 1992). PedsQL, Paediatric Quality of Life Inventory | | | | | | | | |

**Aged 3 years**

| Table C1 – Response distribution, in children aged 3 years | | | | | | |
| --- | --- | --- | --- | --- | --- | --- |
| Dimension | Total sample | General health groups | | | |  |
|  |  | Excellent | Very good | Good | Fair or poor | p-value (Fisher’s) |
| **Movement** |  |  |  |  |  | <0.001 |
| Level 1 | 229 (92.7%) | 91 (100%) | 86 (97%) | 37 (74%) | 15 (88%) |  |
| Level 2 | 13 (5.3%) |  | 2 (2.2%) | 10 (20%) | 1 (5.9%) |  |
| Level 3 | 5 (2%) |  | 1 (1.1%) | 3 (6.0%) | 1 (5.9%) |  |
| **Play** |  |  |  |  |  | <0.001 |
| Level 1 | 218 (88.3%) | 84 (92%) | 83 (93%) | 37 (74%) | 14 (82%) |  |
| Level 2 | 24 (9.7%) | 6 (6.6%) | 6 (6.7%) | 10 (20%) | 2 (12%) |  |
| Level 3 | 5 (2%) | 1 (1.1%) |  | 3 (6.0%) | 1 (5.9%) |  |
| **Pain** |  |  |  |  |  | <0.001 |
| Level 1 | 194 (78.5%) | 84 (92%) | 73 (82%) | 30 (60%) | 7 (41%) |  |
| Level 2 | 52 (21.1%) | 7 (7.7%) | 16 (18%) | 20 (40%) | 9 (53%) |  |
| Level 3 | 1 (0.4%) |  |  |  | 1 (5.9%) |  |
| **Social**  **interaction** |  |  |  |  |  | <0.001 |
| Level 1 | 181 (73.3%) | 80 (88%) | 65 (73%) | 25 (50%) | 11 (65%) |  |
| Level 2 | 52 (21.1%) | 9 (9.9%) | 20 (22%) | 19 (38%) | 4 (24%) |  |
| Level 3 | 14 (5.7%) | 2 (2.2%) | 4 (4.5%) | 6 (12%) | 2 (12%) |  |
| **Communication** |  |  |  |  |  | <0.001 |
| Level 1 | 185 (74.9%) | 79 (87%) | 65 (73%) | 29 (58%) | 12 (71%) |  |
| Level 2 | 50 (20.2%) | 12 (13%) | 20 (22%) | 14 (28%) | 4 (24%) |  |
| Level 3 | 12 (4.9%) |  | 4 (4.5%) | 7 (14%) | 1 (5.9%) |  |
| **Eating** |  |  |  |  |  | <0.001 |
| Level 1 | 143 (57.9%) | 66 (73%) | 50 (56%) | 21 (42%) | 6 (35%) |  |
| Level 2 | 74 (30%) | 23 (25%) | 30 (34%) | 17 (34%) | 4 (24%) |  |
| Level 3 | 30 (12.1%) | 2 (2.2%) | 9 (10%) | 12 (24%) | 7 (41%) |  |
| ceiling effect | 103 (41.7%) | 56 (61.5%) | 36 (40.4%) | 8 (16%) | 3 (17.6%) | <0.001 |
| LSS (mean, SD) | 7.62 ( 2.06 ) | 6.74 (1.19) | 7.46 (1.8) | 9.04 (2.53) | 8.94 (2.63) | <0.001 |
| EQ VAS (mean, SD) | 82.2 ( 15.97 ) | 90.96 (9.8) | 83.71 (11.13) | 74.7 (13.55) | 49.53 (19.39) | <0.001 |
| Level 1: no problems, Level 2: some problems; Level 3: a lot of problems  General health groups were identified based on parents/caregivers response to the general health question to describe their child’s general health today as ‘excellent’, ‘very good’, ‘good’, ‘fair’ or ‘poor’. Based on this question, children were categorized into four groups: 1) excellent, 2) very good, 3) good, 4) fair and poor. | | | | | | |

| Table C2 Known-group validity in children aged 3 years | | | | | | | | | | |
| --- | --- | --- | --- | --- | --- | --- | --- | --- | --- | --- |
|  | EQ-TIPS Level sum scores | | | | | PedsQL total scores | | | | |
| Groups | N | Mean | SD | p value | effect size | N | Mean | SD | p value | effect size |
| **General health** | | | | | | | | | | |
| Excellent | 91 | 6.74 | 1.19 | <0.001 | 0.2 | 91 | 85.84 | 12.15 | <0.001 | 0.17 |
| very good | 89 | 7.46 | 1.8 |  |  | 89 | 78.3 | 16.15 |  |  |
| good | 50 | 9.04 | 2.53 |  |  | 50 | 69.11 | 17.58 |  |  |
| fair or poor | 17 | 8.94 | 2.63 |  |  | 17 | 67.62 | 12.01 |  |  |
| **Special health care needs** | | | | | | | | | | |
| No | 181 | 7.12 | 1.36 | <0.001 | 0.99 | 181 | 82.21 | 13.84 | <0.001 | 0.93 |
| Yes | 66 | 8.98 | 2.89 |  |  | 66 | 68.25 | 18.02 |  |  |
| **Chronic health condition** | | | | | | | | | | |
| No | 167 | 7.14 | 1.36 | <0.001 | 0.76 | 167 | 82.08 | 14.16 | <0.001 | 0.72 |
| Yes | 80 | 8.61 | 2.8 |  |  | 80 | 70.98 | 17.82 |  |  |
| **Eczema** |  |  |  |  |  |  |  |  |  |  |
| No | 68 | 6.69 | 1.1 | <0.001 | 0.81 | 68 | 84.64 | 16.09 | <0.001 | 0.52 |
| Yes | 42 | 7.86 | 1.88 |  |  | 42 | 76.26 | 16.31 |  |  |
| **Sleep problems** |  |  |  |  |  |  |  |  |  |  |
| No | 68 | 6.69 | 1.1 | <0.001 | 1.05 | 68 | 84.64 | 16.09 | <0.001 | 0.92 |
| Yes | 51 | 8.45 | 2.23 |  |  | 51 | 70.11 | 15.55 |  |  |
| **Asthma** |  |  |  |  |  |  |  |  |  |  |
| No | 68 | 6.69 | 1.1 | <0.001 | 0.63 | 68 | 84.64 | 16.09 | <0.001 | 0.5 |
| Yes | 31 | 7.74 | 2.49 |  |  | 31 | 76.34 | 17.66 |  |  |
| **Developmental delay** |  |  |  |  |  |  |  |  |  |  |
| No | 68 | 6.69 | 1.1 | <0.001 | 2.07 | 68 | 84.64 | 16.09 | <0.001 | 1.24 |
| Yes | 24 | 10.38 | 2.98 |  |  | 24 | 64.72 | 16.2 |  |  |
| **Food allergies** |  |  |  |  |  |  |  |  |  |  |
| No | 68 | 6.69 | 1.1 | <0.001 | 1.51 | 68 | 84.64 | 16.09 | <0.001 | 0.67 |
| Yes | 20 | 9.35 | 3.12 |  |  | 20 | 73.89 | 15.83 |  |  |
| **Atopy** |  |  |  |  |  |  |  |  |  |  |
| No | 68 | 6.69 | 1.1 | <0.001 | 0.75 | 68 | 84.64 | 16.09 | <0.001 | 0.54 |
| Yes | 70 | 8.11 | 2.42 |  |  | 70 | 75.77 | 16.86 |  |  |
| **Brain related Disorders** |  |  |  |  |  |  |  |  |  |  |
| No | 68 | 6.69 | 1.1 | <0.001 | 1.71 | 68 | 84.64 | 16.09 | <0.001 | 1.25 |
| Yes | 40 | 9.93 | 2.76 |  |  | 40 | 64.91 | 15.17 |  |  |
| Cohen’s D effect size thresholds 0.2–0.49, 0.5–0.79 and >0.8 denote small, medium and large effect size, respectively.  The healthy group was free of any chronic condition and had an EQ VAS score of at least 70 at baseline.  PedsQL Paediatric Quality of Life Inventory, SD standard deviation | | | | | | | | | | |

| Table C3 Test-retest reliability analysis among children aged 3 years (N=83) | | |
| --- | --- | --- |
| EQ-TIPS dimension |  | Weighted kappa (standard error) |
| Movement | kappa | 0.57(0.07) |
| Play | kappa | 0.45(0.10) |
| Pain | kappa | 0.54(0.10) |
| Social Interaction | kappa | 0.54(0.09) |
| Communication | kappa | 0.67(0.09) |
| Eating | kappa | 0.48(0.08) |
| PedsQL domains |  | ICC (95% Confidence Interval) |
| Physical Functioning | ICC | 0.61(0.46 -0.73) |
| Emotional Functioning | ICC | 0.58(0.42-0.71) |
| Social Functioning | ICC | 0.76(0.65 – 0.84) |
| School Functioning | ICC | 0.62(0.48 – 0.75) |
| Summary score |  |  |
| EQ-TIPS LSS | ICC | 0.86 (0.79 -0.91) |
| PedsQL | ICC | 0.68(0.55-0.78) |
| ICC intraclass correlation coefficient; LSS: level sum score; PedsQL Paediatric Quality of Life Inventory.  At dimension level, Weighted kappa coefficient was calculated. Coefficient values of 0.2, 0.21–0.40, 0.41–0.6, 0.61–0.80 and >0.81 were indicative of poor, fair, moderate, substantial, and almost-perfect agreement, respectively.  ICC is based on comparison of EQ-TIPS LSS, PedsQL domain and total score at initial and follow-up survey for participants who completed follow-up 4 weeks after initial survey and reported no change in health; ICC thresholds of <0.4, 0.4–0.59, 0.60–0.74 and > 0.75 were indicative of poor, fair, good, and excellent reliability, respectively | | |

| Table C4. Correlations between PedsQL item and EQ-TIPS item, among children aged 3 years old | | | | | | | |  |
| --- | --- | --- | --- | --- | --- | --- | --- | --- |
|  | EQ-TIPS | | | | | | |  |
|  | movement | play | pain | social interaction | communication | eating | EQ-TIPS LSS |  |
| **PedsQL Physical functioning** |  |  |  |  |  |  |  |  |
| walking | -0.58 | -0.32 | -0.31 | -0.32 | -0.29 | -0.11 | -0.34 |  |
| running | -0.49 | -0.28 | -0.20 | -0.34 | -0.31 | -0.09 | -0.32 |  |
| active play or exercise | -0.38 | -0.35 | -0.26 | -0.41 | -0.38 | -0.21 | -0.45 |  |
| lifting something heavy | -0.29 | -0.33 | -0.14 | -0.24 | -0.26 | -0.16 | -0.30 |  |
| bathing | -0.29 | -0.34 | -0.29 | -0.28 | -0.35 | -0.30 | -0.46 |  |
| helping to pick up toys | -0.25 | -0.33 | -0.23 | -0.33 | -0.39 | -0.32 | -0.46 |  |
| hurts or aches | -0.23 | -0.27 | -0.45 | -0.19 | -0.21 | -0.26 | -0.41 |  |
| low energy level | -0.28 | -0.25 | -0.26 | -0.25 | -0.28 | -0.21 | -0.37 |  |
| Sub domain score | -0.36 | -0.39 | -0.39 | -0.40 | -0.42 | -0.34 | -0.57 |  |
| **PedsQL Emotional functioning** |  |  |  |  |  |  |  |  |
| afraid or scared | -0.11 | -0.13 | -0.18 | -0.21 | -0.17 | -0.20 | -0.26 |  |
| sad or blue | -0.13 | -0.20 | -0.28 | -0.28 | -0.21 | -0.25 | -0.36 |  |
| angry | -0.08 | -0.17 | -0.19 | -0.28 | -0.24 | -0.32 | -0.40 |  |
| trouble sleeping | -0.13 | -0.20 | -0.32 | -0.23 | -0.23 | -0.31 | -0.40 |  |
| worrying | -0.18 | -0.19 | -0.32 | -0.28 | -0.28 | -0.22 | -0.37 |  |
| Sub domain score | -0.17 | -0.25 | -0.35 | -0.35 | -0.31 | -0.36 | -0.49 |  |
| **PedsQL Social functioning** |  |  |  |  |  |  |  |  |
| playing with other children | -0.22 | -0.29 | -0.23 | -0.47 | -0.37 | -0.17 | -0.41 |  |
| other kids not wanting to play with them | -0.21 | -0.24 | -0.14 | -0.31 | -0.27 | -0.21 | -0.35 |  |
| teased by other children | -0.04 | -0.19 | -0.02 | -0.23 | -0.25 | -0.13 | -0.25 |  |
| not able to do things that other children can do | -0.33 | -0.34 | -0.30 | -0.50 | -0.49 | -0.32 | -0.54 |  |
| keeping up when playing with other children | -0.25 | -0.30 | -0.29 | -0.34 | -0.32 | -0.22 | -0.40 |  |
| Sub domain score | -0.29 | -0.35 | -0.30 | -0.48 | -0.43 | -0.27 | -0.51 |  |
| **PedsQL School functioning** |  |  |  |  |  |  |  |  |
| same school activities as peers | -0.32 | -0.27 | -0.32 | -0.30 | -0.34 | -0.23 | -0.39 |  |
| missing school because of not feeling well | -0.05 | -0.15 | -0.25 | -0.14 | -0.19 | -0.18 | -0.26 |  |
| missing school to go to doctor | -0.24 | -0.27 | -0.21 | -0.26 | -0.31 | -0.21 | -0.33 |  |
| Sub domain score | -0.26 | -0.27 | -0.36 | -0.28 | -0.35 | -0.25 | -0.41 |  |
| **PesQL total score** | -0.34 | -0.39 | -0.41 | -0.47 | -0.46 | -0.37 | -0.62 |  |
| Correlations were calculated using Spearman’s correlation. Correlations of 0.1–0.29 were considered weak, 0.3–0.49 moderate, and ≥ 0.5 strong (Cohen J 1992). PedsQL, Paediatric Quality of Life Inventory | | | | | | | | |
